# Exploring Sentiment and Care Management of Hospitalized Patients During the First Wave of the COVID-19 Pandemic Using Electronic Nursing Health Records: Descriptive Study

**DOI:** 10.2196/38308

**Published:** 2022-05-12

**Authors:** Juan Nicolás Cuenca-Zaldívar, Maria Torrente-Regidor, Laura Martín-Losada, César Fernández-De-Las-Peñas, Lidiane Lima Florencio, Pedro Alexandre Sousa, Domingo Palacios-Ceña

**Affiliations:** 1 Research Group in Nursing and Health Care Puerta de Hierro Health Research Institute - Segovia de Arana Majadahonda Spain; 2 Functional Recovery Unit Guadarrama Hospital Guadarrama Spain; 3 Servicio de Oncología Médica Hospital Universitario Puerta de Hierro Majadahonda Spain; 4 Faculty of Health Sciences Universidad Francisco de Vitoria Majadahonda Spain; 5 Research Group of Manual Therapy Department of Physical Therapy, Occupational Therapy, Physical Medicine, and Rehabilitation Universidad Rey Juan Carlos Alcorcón Spain; 6 Department of Electrical Engineering Faculty of Science and Technology Universidade Nova de Lisboa Lisbon Portugal; 7 Research Group of Humanities and Qualitative Research in Health Science Department of Physical Therapy, Occupational Therapy, Physical Medicine and Rehabilitation Universidad Rey Juan Carlos Alcorcón Spain

**Keywords:** electronic health records, COVID-19, pandemic, content text analysis

## Abstract

**Background:**

The COVID-19 pandemic has changed the usual working of many hospitalization units (or wards). Few studies have used electronic nursing clinical notes (ENCN) and their unstructured text to identify alterations in patients' feelings and therapeutic procedures of interest.

**Objective:**

This study aimed to analyze positive or negative sentiments through inspection of the free text of the ENCN, compare sentiments of ENCN with or without hospitalized patients with COVID-19, carry out temporal analysis of the sentiments of the patients during the start of the first wave of the COVID-19 pandemic, and identify the topics in ENCN.

**Methods:**

This is a descriptive study with analysis of the text content of ENCN. All ENCNs between January and June 2020 at Guadarrama Hospital (Madrid, Spain) extracted from the CGM Selene Electronic Health Records System were included. Two groups of ENCNs were analyzed: one from hospitalized patients in post–intensive care units for COVID-19 and a second group from hospitalized patients without COVID-19. A sentiment analysis was performed on the lemmatized text, using the National Research Council of Canada, Affin, and Bing dictionaries. A polarity analysis of the sentences was performed using the Bing dictionary, SO Dictionaries V1.11, and Spa dictionary as amplifiers and decrementators. Machine learning techniques were applied to evaluate the presence of significant differences in the ENCN in groups of patients with and those without COVID-19. Finally, a structural analysis of thematic models was performed to study the abstract topics that occur in the ENCN, using Latent Dirichlet Allocation topic modeling.

**Results:**

A total of 37,564 electronic health records were analyzed. Sentiment analysis in ENCN showed that patients with subacute COVID-19 have a higher proportion of positive sentiments than those without COVID-19. Also, there are significant differences in polarity between both groups (*Z*=5.532, *P*<.001) with a polarity of 0.108 (SD 0.299) in patients with COVID-19 versus that of 0.09 (SD 0.301) in those without COVID-19. Machine learning modeling reported that despite all models presenting high values, it is the neural network that presents the best indicators (>0.8) and with significant *P* values between both groups. Through Structural Topic Modeling analysis, the final model containing 10 topics was selected. High correlations were noted among topics 2, 5, and 8 (pressure ulcer and pharmacotherapy treatment), topics 1, 4, 7, and 9 (incidences related to fever and well-being state, and baseline oxygen saturation) and topics 3 and 10 (blood glucose level and pain).

**Conclusions:**

The ENCN may help in the development and implementation of more effective programs, which allows patients with COVID-19 to adopt to their prepandemic lifestyle faster. Topic modeling could help identify specific clinical problems in patients and better target the care they receive.

## Introduction

On March 11, 2020, the World Health Organization declared COVID-19 a global pandemic [1]. SARS-CoV-2 presented a great capacity for contagion, spread, and high mortality, which collapsed health care systems worldwide [2,3]. Owing to the sudden spread of the virus, health care professionals have undergone a huge, rapid, and profound change in their professional workplace to combat COVID-19.

In this context, receiving a diagnosis of COVID-19 and being admitted to hospital, often in intensive care units for periods of weeks or even months, provoked a sense of helplessness and near death. This situation has led to an increased prevalence of mental health problems owing to a high rate of prevalence of anxiety and depression among patients with COVID-19 [4], which approached 30% [5] and led to posttraumatic stress in up to 96.2% of those affected [6]. Medical activity has focused primarily on the treatment of the disease [7] and research has focused on epidemiological [8,9], clinical, and pathophysiological factors [10,11].

During the COVID-19 pandemic, electronic health records (EHRs) have provided an agile response to the needs of health care workers and researchers through useful data exploitation [12,13] by presenting information quickly and efficiently, for primary and secondary uses in clinical care [14]. This system allowed having complete and coherent information regardless of where or by whom it was generated, enabling it to follow the timeline of the patient’s disease, including symptoms, acute events, or changes in their treatment or health status [15], which was especially key given the high rotation of health care workers. On the other hand, a fundamental point in EHRs is correct recording of the information in order to be able to make effective and safe clinical decisions for the patient [16]. Previous studies show how the lack of registration of information on the diagnostic process, identification, and listing of events on care and treatment can affect the monitoring of the quality, safety, and efficacy of health care interventions [17]. These clinical notes can be only written by EHR users responsible for patient care, such as doctors, nurses, and assistant nurses [18,19]. Wisner et al [20] showed that the absence or limitation in nursing clinical narratives, comments, and clinical notes hinders clinical reasoning and decision-making along with the transmission of information between the different shifts.

Electronic nursing clinical notes (ENCN) are documents in which nurses describe health status, nursing care, medication, and other observations about patients [21]. In these texts, they also describe their observations and opinions in an attempt to better understand the patients' condition and opinions [22] and among them, the feelings perceived during their interaction [21]. The appropriate use of ENCN can help improve both physical and mental health care of hospitalized patients [23].

Much of the relevant information is recorded in ENCN in the form of free text (unstructured), known as clinical notes, which makes analysis and decision-making very difficult. This has stimulated the development of semantic analysis methods [24,25] that allow in-depth exploration of the clinical information potentially available in health services [26] and determine the amount of information collected about a clinical process or condition [18-20] and the content of that information regarding specific topics.

Sentiment or opinion analysis allows the analysis of positive or negative sentiments in a text by using precalibrated dictionaries of terms [27]. Polarity facilitates the qualification of these sentiments in the context of sentences; for example, the term “happy” denotes a clearly positive sentiment, but if it is preceded by “not happy” in the sentence, the polarity is reversed toward a negative value [28]. Sentiment analysis in the health care domain has been used in the analysis of social networks [29,30], suicide notes [31], or radiology notes [32], as well as nursing notes [33]. The application of this type of analysis provides insight into patients' attitudes toward the contextual polarity of ENCN and assesses symptoms related to their mental health, which may not have been detected through direct analysis [22].

Latent Dirichlet Allocation (LDA) thematic pattern analysis is a technique to detect hidden topics in a corpus of texts [34]. It assumes topics with word clusters in which the distribution of words within each topic is taken into account, along with the distribution of topics throughout the corpus [35]. This technique has been used in social network analysis, news [36,37], or in response to government policies [38]. Biomedical terms have been found to form specific topics [39,40]; so, this analysis can provide useful clinical information [35].

To our best of knowledge, there are currently no studies describing the use of ENCN for the determination of sentiment and polarity (rejection-acceptance) as well as the identification of clinical practices of interest of hospitalized patients during the start of the first wave of the COVID-19 pandemic.

Therefore, the objectives of this study were the following: (1) analysis of patient´s sentiments through the analysis of the free text of the ENCN, (2) comparison of the sentiments and polarity of hospitalized patients in post–intensive care units for COVID-19 with those hospitalized in non–COVID-19 wards, (3) temporal analysis of the patients´ sentiments during the first wave of the pandemic (January to June 2020) through the ENCN, and (4) identification of the contents and topics that appear in the ENCN.

## Methods

### Design

This is a descriptive study that involves an analysis of the textual content of the ENCN [41]. Through the analysis of narrative texts, the positive and negative sentiments of patients can be described and analyzed [42]. The object of the textual analysis studies is to understand how a certain event affects the attitudes and behaviors of people. This study focuses on the ENCN of the nurses who worked during an outbreak of the COVID-19 pandemic in a Spanish hospital [41].

### Ethical Considerations

This study was approved by the Clinical Research Ethics Committee at the Hospital Universitario Puerta de Hierro Majadahonda de Madrid (07/400080.9/22). Also, for reviewing clinical histories and data, we had approval from the Guadarrama Hospital Center Management. At all times, the confidentiality of the information was preserved, thus ensuring responsible use of the data, as established by current Spanish regulations and in accordance with the tenets of the Declaration of Helsinki.

### Setting, Sample, and Data Collection Tools

All clinical notes contained in the ENCN registered between January and June 2020 at Guadarrama Hospital were extracted from the CGM Selene EHR System (CompuGroup Medical Deutschland AG). Guadarrama Hospital is a mid-term stay hospital in the Community of Madrid, with 144 beds, and provides rehabilitation and long-term care to patients with chronic pathologies; however, during the COVID-19 pandemic, it also provided care to patients with a COVID-19 infection.

The analyzed records collect follow-up data from the day of admission until discharge or death, collecting up to 3 records per day in each work shift (morning shift from 8 AM to 3 PM, afternoon shift from 3 PM to 10 PM, and night shift from 10 PM to 8 AM). ENCN from two groups of nurses were analyzed: one from nurses working with hospitalized post–intensive care unit patients with COVID-19 and a the other from nurses working in non–COVID-19 wards. The hospital´s physicians diagnosed and confirmed COVID-19 and assigned patients to the different wards.

### Statistical Analysis

For the statistical analysis, the R package (version 3.5.1; R Foundation for Statistical Computing) was used. The level of significance was established at *P*<.05.

#### Sentiment Analysis

Previously, the text was standardized by lemmatizing it and cleaning up the stop words. A sentiment analysis was performed on the text using the National Research Council of Canada’s (NRC’s) Emotion Lexicon [43], Affin [44], and Bing [45] dictionaries. All three of these lexicons are based on unigrams or single Spanish words that assign scores for positive or negative sentiment. In addition, the NRC dictionary categorizes words into emotional categories of anger, anticipation, disgust, fear, joy, sadness, surprise, and trust, while the Affin lexicon assigns words with a score between –5 and +5, with negative values indicating negative sentiment and positive values indicating positive sentiment. The presence of significant differences between ENCN in groups of patients with and those without COVID-19 was verified using the Pearson chi-square test, with Bonferroni correction for post hoc analysis. The temporal evolution of sentiments in both groups was evaluated using the Dynamic Time Warp test, which allows comparing time series of different lengths using the normalized Euclidean distance.

#### Polarity Analysis

In addition, a polarity analysis (Textboxes 1 and 2) of the sentences was performed using the Bing dictionary, the SO Dictionaries V1.11, and Spa [46-48] dictionary as amplifiers and decrementators, and those proposed by Vilares et al [49] as deniers. The Mann-Whitney *U* test was used between the two groups of patients to test significant differences after verifying the nonnormal distribution of polarity using the Kolmogorov-Smirnov test with Lilliefors correction.

**The polarity calculation process.**

Four phases were used progressively for the analysis of acceptance-rejection (polarity):

**Phase 1.** We created a file with the text of the interviews broken down by phrases for textual analysis.

**Phase 2.** We calculated polarity using the Bing Sentiment Dictionary, the amplifiers and deamplifiers from SO Dictionaries V1.11 and Spa, and the negators proposed by Vilares et al [49].

**Phase 3.** We calculated the scatterplot of the sentences in the text regarding neutrality to identify positive or negative trends.

**Phase 4.** The evolution of the emotional valence (positive-negative) would be shown throughout the interviews. We applied Fourier transformation to confirm the polarity trend.

**Formula and dictionaries used to calculate polarity.**

The analysis was carried out using the Bing dictionary [28]. The Bing dictionary determines the positivity (acceptance) or negativity (rejection) of each word used. Also, the amplifiers and deamplifiers of SO Dictionaries V1.11 and Spa dictionary [29-31] were used, along with negators proposed by Denecke et al [32].

To calculate the polarity (δ), a context cluster of words (x^T^_i_ ) is formed around each polarized word using the Bing dictionary [28], taking by default 4 words before and 2 words after it (if there is any comma in the cluster, it will only include the words that are after the comma), and those will be treated as valence shifters.

The words in this cluster are labeled as neutral (x^0^_i_), negators (x^N^_i_), amplifiers (x^a^_i_) or de-amplifiers (x^d^_i_) using the dictionary SO Dictionaries V1.11 Spa2 [47] and the negators proposed by Hu and Liu [48]. Neutral words do not add to the equation but affect the word count (n).

Each polarized word (negative or positive) is weighted (w) on the basis of the context cluster weights (x^T^_i_) and further weighted by the number and position of the valence shifters directly surrounding it. A weight (c) can be added and applied to both amplifiers and deamplifiers (with a default value of 0.8 and a lower limit for the deamplifiers of –1).

Finally, the context cluster (x^T^_i_) is added and divided by the square root of the number of words (√n) to generate a polarity score (δ) that, by default, is not limited in value.

The final result is the following formula:

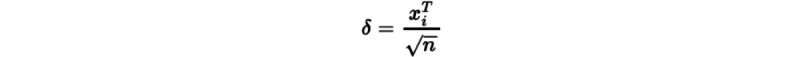

Where:

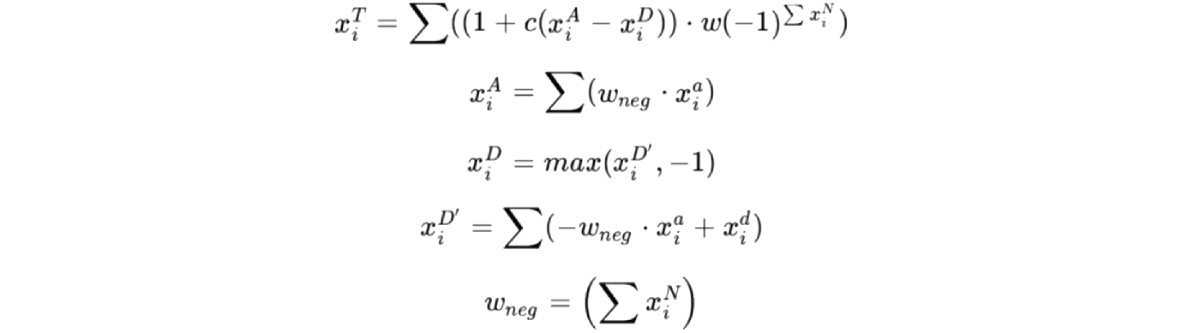

#### ENCN Comparison

Machine learning enables the automation of large amounts of text by model training [50]. Machine learning techniques were applied in order to evaluate the presence of significant differences in the ENCN among patients with and those without COVID-19. For this, the models were created on a random subsample of 75% of the text, applying them to the remaining 25%. The applied models were Support Vector Machine, Naive-Bayes, random forest, and neural network. The quality of the models was evaluated using the area under the curve (AUC), sensitivity and specificity, the κ index, and accuracy with its level of significance. Values above 0.8 and significant *P* values (*P*<.05) were considered the cutoff point.

#### Topic and Content Analysis

A structural analysis of thematic models (STM) was performed to study the abstract topics that occur in the comments, using LDA topic modeling but allows their inclusion as covariates in the model, the temporal evolution, and the presence of the of ENCN in groups of patients with subacute COVID-19 and those without COVID-19 [33]. The optimal number of topics was determined while considering exclusivity [34] and semantic coherence [35] as criteria. Exclusivity evaluates if the top words for the topics appear within top words of other topics, while semantic coherence shows if the words that are most associated with the corresponding themes occur equally within the documents; in both cases, higher values are better. The effect of the topics of the final model between ENCN in patients with and those without COVID-19 was analyzed, along with the temporal evolution in the prevalence of the appearance of global themes between both groups. The interaction graph was used to determine the presence of significant differences in the evolution of prevalence between both groups. An analysis of the content of the topics and the differences in themes between both groups was carried out, while the network graph allowed for the detection of the presence of categories between topics.

## Results

A total of 37,564 records were analyzed, after eliminating 24,101 duplicates (ie, ENCN that had been copied and pasted from previous ones). ENCN were produced by 77 nurses distributed by working shift, hospital unit, and months (Table 1).

These records correspond to 710 patients, whose baseline demographics and clinical data are shown depending on whether or not they were infected with SARS-CoV-2 (sociodemographic data in Multimedia Appendix 1).

**Table 1:** Distribution of electronic clinical nursing notes by working shift, units, and time (in months).

		Electronic nursing clinical notes for the COVID-19 group, n (%)	Electronic nursing clinical notes for the non–COVID-19 group, n (%)
**Working shift**			
	Morning	5161 (13.7)	10,791 (28.7)
	Afternoon	3637 (9.6)	7931 (21.1)
	Night	3992 (10.6)	6050 (16.1)
**Month**			
	January	161 (0.4)	7360 (19.5)
	February	466 (1.2)	6225 (16.5)
	March	2457 (6.5)	4104 (10.9)
	April	5467 (14.5)	396 (1.0)
	May	3245 (8.6)	2093 (5.5)
	June	994 (2.6)	4594 (12.2)

### Sentiment Analysis

The differences in the sentiments expressed in the ENCN between both groups were significant in the NRC dictionary (*χ*^2^_9_=360.6, *P*<.001), Afinn lexicon both in the scores (*χ*^2^_8_=385.3, *P*<.001) and polarity (*χ*^2^_1_=232.7, *P*<.001), and Bing dictionary (*χ*^2^_1_=368.9, *P*<.001). Post hoc tests showed significant differences among all levels (Multimedia Appendices 2 and 3).

In the ENCN of patients with COVID-19, there is a higher proportion of positive sentiments than that in the non–COVID-19 group. The most frequently expressed emotion is sadness, which was greater in the non–COVID-19 group, followed by trust, which appears to be similar in both groups. Sentiments with negative scores (–2) are more frequent in the non–COVID-19 group, while that of positive sentiments was higher in the COVID-19 group (+2) (Table 2).

The evolution of the sentiments expressed in the ENCN was similar in both groups, revealing a drastic reduction during April and May in the non–COVID-19 group, consistent with the peak of the pandemic (Multimedia Appendix 4).

However, higher values were generally observed in the sentiments expressed in the COVID-19 group when they were analyzed with the Afinn dictionary, where the emotional valences doubled those of patients without COVID-19 and where we observed a clear asymmetry in the distribution of the most negative sentiments (scores of –5).

The distances between both time series are generally small; that is, <0.2. The NRC dictionary showed the greatest differences between the 2 groups in the emotions of surprise and sadness, in the positive sentiments of the Bing dictionary, and in the negative ones of the Afinn dictionary (Multimedia Appendix 5).

**Table 2:** Sentiment scores by dictionary.

Dictionaries		Electronic nursing clinical notes for the COVID-19 group, mean	Electronic nursing clinical notes for the non–COVID-19 group, mean	*P* value
**National Research Council of Canada dictionary**				<.001
	Anger	2.8	2.7	
	Anticipation	8.7	8.5	
	Disgust	3.3	3.9	
	Fear	6.6	7.3	
	Joy	5.2	4.6	
	Sadness	17.1	18.0	
	Surprise	3.6	2.9	
	Trust	10.6	10.4	
	Negative	20.0	21.3	
	Positive	22.0	20.6	
**Afinn dictionary**				<.001
	–5	0.0	0.0	
	–4	0.1	0.1	
	–3	2.1	1.7	
	–2	36.0	43.4	
	–1	18.7	18.3	
	+1	6.2	6.6	
	+2	36.0	29.2	
	+3	0.9	0.7	
	+4	0.0	0.0	
**Afinn dictionary** **(positive-negative)**				<.001
	Negative	56.9	63.5	
	Positive	43.1	36.5	
**Bing dictionary**				<.001
	Negative	34.3	40.4	
	Positive	65.7	59.6	

### Polarity Analysis

Polarity scores were nonnormally distributed between the COVID-19 and non–COVID-19 groups (*P*<.001).

There are significant differences in polarity between both groups (*Z*=5.532, *P*<.001): 0.108 (SD 0.299) in patients with COVID-19 versus 0.09 (SD 0.301) in those without COVID-19.

**Figure 1:**
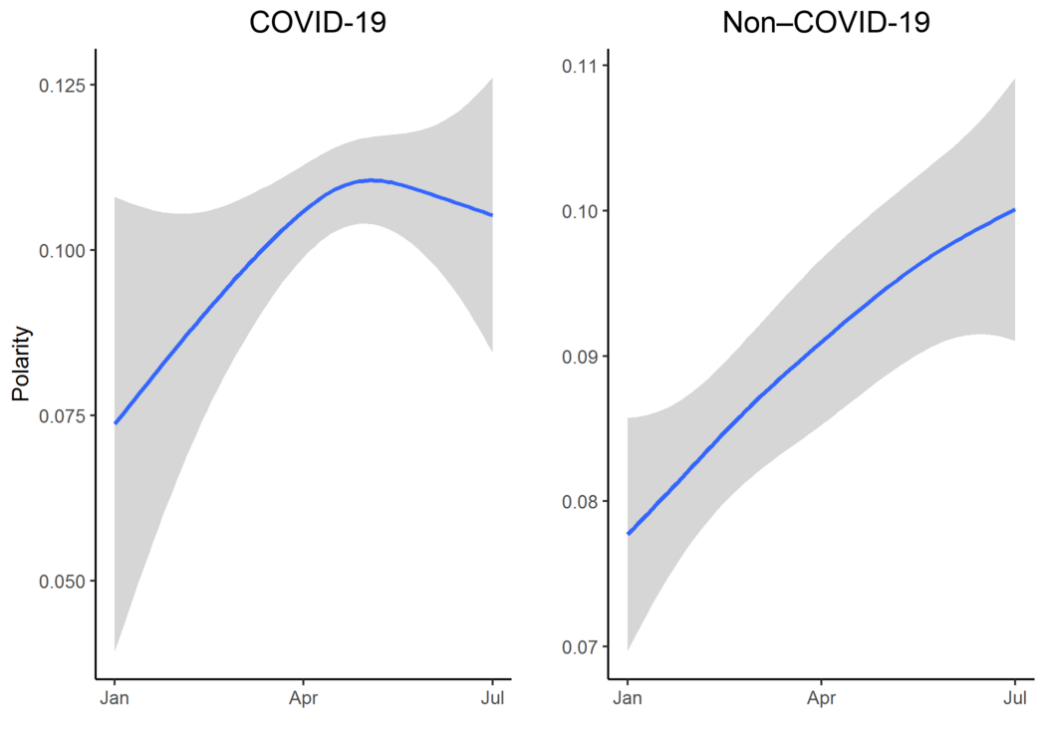
Polarity of patients' comments.

When both groups were compared, we verified how the polarity presents a clear upward trend in ENCN of the non–COVID-19 group, while in ENCN of the COVID-19 group, the most positive value was attained in April to decrease later with higher values than those of the non–COVID-19 group (Figure 1).

### STM

**Figure 2:**
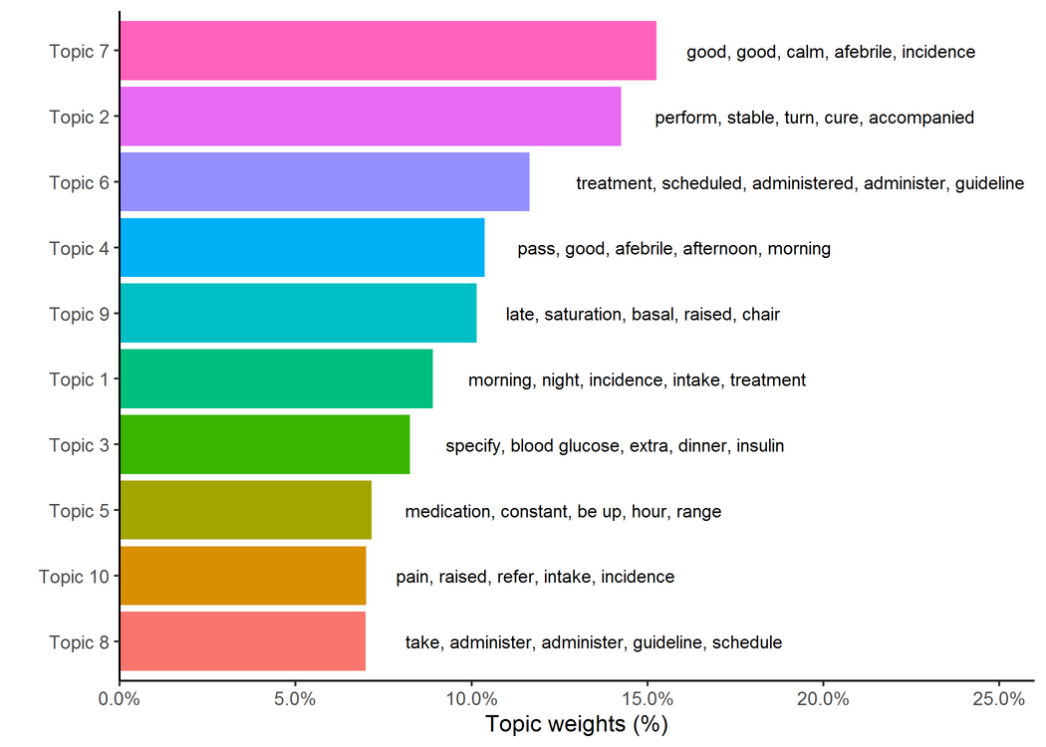
Topic weights and the 5 most frequent words by topic.

The selected model contains 10 topics. The topics tend to be assigned to a few comments, which indicates a high specificity in their content. The presence of the following concepts was hypothesized on the basis of the selected topic weights (see Textbox 3 and Figure 2)

**Topics identified from electronic nursing clinical notes.**

Topic 1: Incidents in each working shift.

Topic 2: Application of pressure ulcer treatments.

Topic 3: Blood glucose level and insulin pattern.

Topic 4: Presence or absence of fever in relation to general condition.

Topic 5: Pharmacotherapy treatment and vital signs control.

Topic 6: Administration of the treatment schedule.

Topic 8: Taking the medication.

Topic 7: Incidents that affect the general well-being of the patient.

Topic 9: Baseline oxygen saturation.

Topic 10: Incidents related to the appearance of pain.

An increase in the prevalence of topics was observed in the second half of the semester, which coincided with the time of admission of patients with COVID-19. Over time, patients with COVID-19 showed a higher prevalence of items 9 (baseline oxygen saturation) and 7 (general well-being), with a lower proportion of items 2 (pressure ulcer treatments), 3 (insulin), 5 (drug therapy and vital sign control), and 10 (pain control) than those without COVID-19. Our findings reported that the main problems among patients with COVID-19 were related to initial oxygen saturation and general well-being, while in those without COVID-19, problems were related to pressure ulcer treatment, pain, diabetes, and drug therapy. The analysis shows different nuances between patients with and those without COVID-19 in the topics of the model. Control of baseline oxygen saturation, blood glucose level, and ingestion, as well as fever, are of greater importance to patients with COVID-19; while among those without COVID-19, pain, insulin dose, pressure ulcer treatment, and pharmacotherapy were the priority topics. In both groups, there is a common concern for the general condition and well-being of the patients, as well as for the control of the treatment regimen.

These differences are significant between both groups over time, as shown in the interaction graph, with an increase in the proportion of topics in the second half of the semester in the COVID-19 group, while in the first half of the semester, this proportion is higher in the non–COVID-19 group. Topics 9 (baseline oxygen saturation) and 2 (pressure ulcer treatment) present the greatest and significant effects between both groups, while topics 8 and 1 do not show any significant effect.

There was a high correlation among topics 2, 5, and 8 (pressure ulcer care, vital sign control, and pharmacotherapy treatment), topics 1, 4, 7, and 9 (incidences related to working shift, fever and well-being state, and baseline oxygen saturation), and topics 3 and 10 (blood glucose level and pain), while topic 6 (administration of treatment schedule) remains uncorrelated.

### Machine Learning Modeling

Although all the models show high values, the neural network showed the best indicators (>0.8) and with significant *P* values. The worst model was the random forest model, which was clearly overfitting (Table 3).

This result coincides with the findings of the thematic model analysis and may indicate significant differences in the type of nursing comment based on the presence or absence of a COVID-19 infection, with the neural network showing excellent values of sensitivity and specificity, as well as precision.

**Table 3:** Machine learning models quality.

Model	Area under the curve	Sensitivity	Specificity	Accuracy (95% CI)	Accuracy (*P* value)
Support vector machine	0.70	0.91	0.50	0.77 (0.76-0.78)	<.001
Random forest	1.0	1.0	1.0	1 (1-1)	<.001
Naive-Bayes	0.80	0.78	0.82	0.79 (0.79-0.80)	<.001
Neural network	0.96	0.99	0.92	0.97 (0.96-0.97)	<.001

## Discussion

### Principal Findings

Our findings report a higher proportion of positive sentiments among patients with subacute COVID-19 than that of those without COVID-19. Groups also differed on the polarity of their narratives (*P*<.001). Among the machine learning models, the neural network presented the best indicators. In addition, the final STM containing 10 topics with high correlations among topics 2, 5, and 8 (pressure ulcer and pharmacotherapy treatment), topics 1, 4, 7, and 9 (incidences related to fever and well-being state, and baseline oxygen saturation), and topics 3 and 10 (blood glucose level and pain).

Previous studies show the presence of positive sentiments during the pandemic, reflected in gratitude toward health care workers and community support for vulnerable people [51]. Our results show a higher proportion of positive sentiments in the ENCN of the COVID-19 group than that of the non–COVID-19 group. These results are consistent with those reported by Sahoo et al [52] as the patients had been in the intensive care unit for more than 40 days. The authors suggest that patients tend to become progressively more relaxed and that the experience of the ward environment changes, with situations perceived as positive becoming more frequent. The emotion most frequently expressed in ENCN was sadness, which was observed in the non–COVID-19 group. Most patients without COVID-19 were in the functional recovery unit—these patients are characterized as being older adults with a prolonged hospital stay and with comorbidities often associated with physical pain. The feeling of sadness could be related to physical pain, according to Shirai and Soshi [53]. Age is also considered a predisposing factor according to Wu et al [54], where hospitalized older adults are at a higher disposition to sadness.

Among the 10 main topics of the model selected for ENCN, the topics with the greatest weight were the application of treatments for pressure cutaneous lesions in the non–COVID-19 group and baseline oxygen saturation in the COVID-19 group. In both groups of patients, there was a common concern for the general condition and well-being of the patients, as well as for control on the treatment regimen. The relevant issues detected in the ENCN in the COVID-19 group were the stability of vital signs (fever and oxygen saturation), glucose control, and diet. The importance of oxygen saturation is justified by the respiratory involvement by SARS-CoV-2 infection [55]. Glucose control could be explained by its relationship with diabetes mellitus being a metabolic syndrome considered as high risk with respect to COVID-19 severity; it may also be related to the use of corticosteroids for the anti-inflammatory treatment of respiratory infection [56]. Regarding diet, the frequency of ENCN could be associated with irregular or low intakes due to the acute phase, with anosmia and ageusia being typical symptoms of SARS-CoV-2 infection [55].

In the ENCN in the non–COVID-19 group, the presence of skin lesions as a topic of interest could be explained by the prevalence of dependence in hospitalized patients, the rate of which is 8.7% in Spain. Furthermore, pressure injuries account for 7%, according to the fifth Spanish National Study of Prevalence of pressure ulcers and other chronic wounds [57]. In addition, patients in the non–COVID-19 group present risk factors for skin lesions, such as advanced age, comorbidity, prolonged hospitalization, functional limitations, and urinary incontinence [58]. Other topics of interest in the ENCN for the non–COVID-19 group were insulin dose and pharmacotherapy. The presence of comorbidities, such as diabetes mellitus, is a common concern for nurses in both groups. In Spain, this disease has a prevalence of 12.5% in adults, mostly affecting older adults [59]. Other records referred to the assessment and control of pain, a symptom that is usually associated with rehabilitation processes [56,60].

Text analysis of unstructured ENCNs has been used with success previously to determine the quality of the registry [61] and in other unstructured texts such as patient experience [62]. This type of analysis is considered useful to capture the perception of an event, demonstrating reliability in health sciences and COVID-19 issues [51,62]. The ability to identify new topics of interest and detect areas for improvement is also considered important [63]. Regarding the dictionaries used in this study, all of them (NRC, Affin, and Bing) yielded significant results; hence, the selected words can be considered sensitive and useful in the care of patients with and those without COVID-19.

The application of text mining techniques on clinical text may be a valid source for evaluating the sentiments of hospitalized patients and detecting problems related to their mental health (anxiety, depression, and posttraumatic stress), which may influence the evolution of their illness. These results may help establish early and more effective recovery programs that address these issues and allow those affected to return more quickly to their prepandemic lifestyle.

Finally, topic modeling has made it possible to obtain relevant clinical information from the clinical notes, allowing the identification of clinical problems in providing care to patients with and those without COVID-19, which are clearly differentiated, and which may help guide their care more effectively.

### Limitations

This study has limitations. The main outcome could not be compared more broadly owing to the absence of studies on polarity and sentiment in ENCN during the start of first wave of the COVID-19 pandemic. The patients´ sentiments before and during the pandemic could be different; hence, the results of the comparisons between patients with and those without COVID-19 must be interpreted with caution.

### Conclusions

ENCN can provide very useful real-time information, identifying the patient’s sentiments and their polarity (rejection-acceptance). Additionally, it may serve to identify relevant issues based on the care of different groups of patients, both with and those without COVID-19. This can present an opportunity to direct health care strategies in accordance with the needs detected in hospitalized patients, based on real word data, and may help develop and implement preventive programs.

## Appendix

Multimedia Appendix 1 Sociodemographic data.

Multimedia Appendix 2 Significant differences between sentiments. Post hoc pairwise comparison with the NRC dictionary.

Multimedia Appendix 3 Significant differences between all emotional levels. Post hoc pairwise comparison with the Afinn dictionary.

Multimedia Appendix 4 Emotions evolution during first semester of 2020.

Multimedia Appendix 5 Distances between the time series of ENCN of Covid and non-Covid patients.

## Abbreviations

AUC: area under the curve
EHR: electronic health record
ENCN: electronic nursing clinical notes
LDA: Latent Dirichlet Allocation
NRC: National Research Council of Canada
STM: structural analysis of thematic models
SVM: Support Vector Machine
