# Supplementary material for: Exploring Sentiment and Care Management of Hospitalized Patients During the First Wave of the COVID-19 Pandemic Using Electronic Nursing Health Records: Descriptive Study
Source: JMIR Med Inform. 2022 May 12;10(5):e38308. doi: 10.2196/38308 (PMC9106279; doi:10.2196/38308)
Supplement: Multimedia Appendix 1 [file medinform_v10i5e38308_app1.docx]

Multimedia Appendix 1. Sociodemographic data.

|  |  | Patients with COVID-19 | Patients without COVID-19 |
| --- | --- | --- | --- |
| N total |  | 436 | 274 |
| **Sex** |  |  |  |
|  | Male | 196 (45.0%) | 149 (54.4%) |
|  | Female | 240 (55.0%) | 125 (45.6%) |
| **Age** |  |  |  |
|  |  | 77.46±12.45 | 79.32±11.42 |
| **Days of admission** |  |  |  |
|  |  | 43.07±40.14 | 33.53±39.84 |
| **Diagnosis** |  |  |  |
|  | Stroke | 70 (16%) | 11 (4%) |
|  | Palliative care | 68 (15%) | 5 (1%) |
|  | Amputation | 17 (3%) | 2 (0.7%) |
|  | Functional deterioration post-ICU ^a^ | 41 (9%) | 159 (58.0%) |
|  | COPD ^b^ | 15 (3%) | 6 (2%) |
|  | Hip fracture | 119 (27.3%) | 18 (6%) |
|  | Heart failure | 11 (2%) | 2 (0.7%) |
|  | Immobility | 23 (5%) | 1 (0.4%) |
|  | Pneumonia | 22 (5%) | 57 (20%) |
|  | Polineuropathy | 9 (2%) | 0 (0.0%) |
|  | Tetraparesis | 3 (0.7%) | 1 (0.4%) |
|  | Cures for pressure ulcers | 38 (8%) | 12 (4%) |

^a^ ICU: Intensive care unit.

^b^ COPD: Chronic obstructive pulmonary disease.

Data expressed with mean±standard deviation or with absolute and relative values (%).
