# Supplementary material for: Exploring Sentiment and Care Management of Hospitalized Patients During the First Wave of the COVID-19 Pandemic Using Electronic Nursing Health Records: Descriptive Study
Source: JMIR Med Inform. 2022 May 12;10(5):e38308. doi: 10.2196/38308 (PMC9106279; doi:10.2196/38308)
Supplement: Multimedia Appendix 2 [file medinform_v10i5e38308_app2.docx]

Multimedia Appendix 2. Significant differences between sentiments. Post hoc pairwise comparison with the NRC dictionary

| p <- pairwiseNominalIndependence(table(sentnrc$sentiment, sentnrc$COVID), fisher = FALSE, gtest = FALSE, chisq = TRUE, method = "bonferroni")  p1 <- p[p$p.adj.Chisq < 0.05,]  p1$p.adj.Chisq <- round(p1$p.adj.Chisq)  p1 <- p1[c(1, 3)]  p1$p.adj.Chisq <- gsub("^0$", "<0.001", p1$p.adj.Chisq)  kable(na.omit(p1), "html", row.names = FALSE, col.names = c("Comparison", "p value\n(Bonferroni)")) %>% kable_styling(full_width = F) |
| --- |

| **Comparison** | ***P* value (Bonferroni)** |
| --- | --- |
| anger : disgust | <.001 |
| anger : fear | <.001 |
| anger : sadness | <.001 |
| anger : surprise | <.001 |
| anger : negative | <.001 |
| anticipation : disgust | <.001 |
| anticipation : fear | <.001 |
| anticipation : joy | <.001 |
| anticipation : sadness | <.001 |
| anticipation : surprise | <.001 |
| anticipation : negative | <.001 |
| disgust : joy | <.001 |
| disgust : sadness | <.001 |
| disgust : surprise | <.001 |
| disgust : trust | <.001 |
| disgust : negative | <.001 |
| disgust : positive | <.001 |
| fear : joy | <.001 |
| fear : surprise | <.001 |
| fear : trust | <.001 |
| fear : positive | <.001 |
| joy : sadness | <.001 |
| joy : trust | <.001 |
| joy : negative | <.001 |
| sadness : surprise | <.001 |
| sadness : trust | <.001 |
| sadness : positive | <.001 |
| surprise : trust | <.001 |
| surprise : negative | <.001 |
| surprise : positive | <.001 |
| trust : negative | <.001 |
| trust : positive | <.001 |
| negative : positive | <.001 |
