# Supplementary material for: Exploring Sentiment and Care Management of Hospitalized Patients During the First Wave of the COVID-19 Pandemic Using Electronic Nursing Health Records: Descriptive Study
Source: JMIR Med Inform. 2022 May 12;10(5):e38308. doi: 10.2196/38308 (PMC9106279; doi:10.2196/38308)
Supplement: Multimedia Appendix 3 [file medinform_v10i5e38308_app3.docx]

Multimedia Appendix 3. Significant differences between all emotional levels. Post hoc pairwise comparison with the Afinn dictionary

| p <- pairwiseNominalIndependence(table(sentafinn$Puntuacion, sentafinn$COVID), fisher = FALSE, gtest = FALSE, chisq = TRUE, method = "bonferroni")  p2 <- p[p$p.adj.Chisq < 0.05,]  p2$p.adj.Chisq <- round(p2$p.adj.Chisq)  p2 <- p2[c(1, 3)]  p2$p.adj.Chisq <- gsub("^0$", "<0.001", p2$p.adj.Chisq)  kable(na.omit(p2), "html", row.names = FALSE, col.names = c("Comparison", "p value\n(Bonferroni)")) %>% kable_styling(full_width = F) |
| --- |

| **Comparison** | ***P* value (Bonferroni)** |
| --- | --- |
| -3 : -2 | <.001 |
| -3 : 1 | <.001 |
| -2 : -1 | <.001 |
| -2 : 1 | <.001 |
| -2 : 2 | <.001 |
| -2 : 3 | <.001 |
| -1 : 2 | <.001 |
| 1 : 2 | <.001 |
