# Supplementary figures and images for: Exploring Sentiment and Care Management of Hospitalized Patients During the First Wave of the COVID-19 Pandemic Using Electronic Nursing Health Records: Descriptive Study
Source: JMIR Med Inform. 2022 May 12;10(5):e38308. doi: 10.2196/38308 (PMC9106279; doi:10.2196/38308)

Multimedia Appendix 4. Emotions evolution during first semester of 2020.


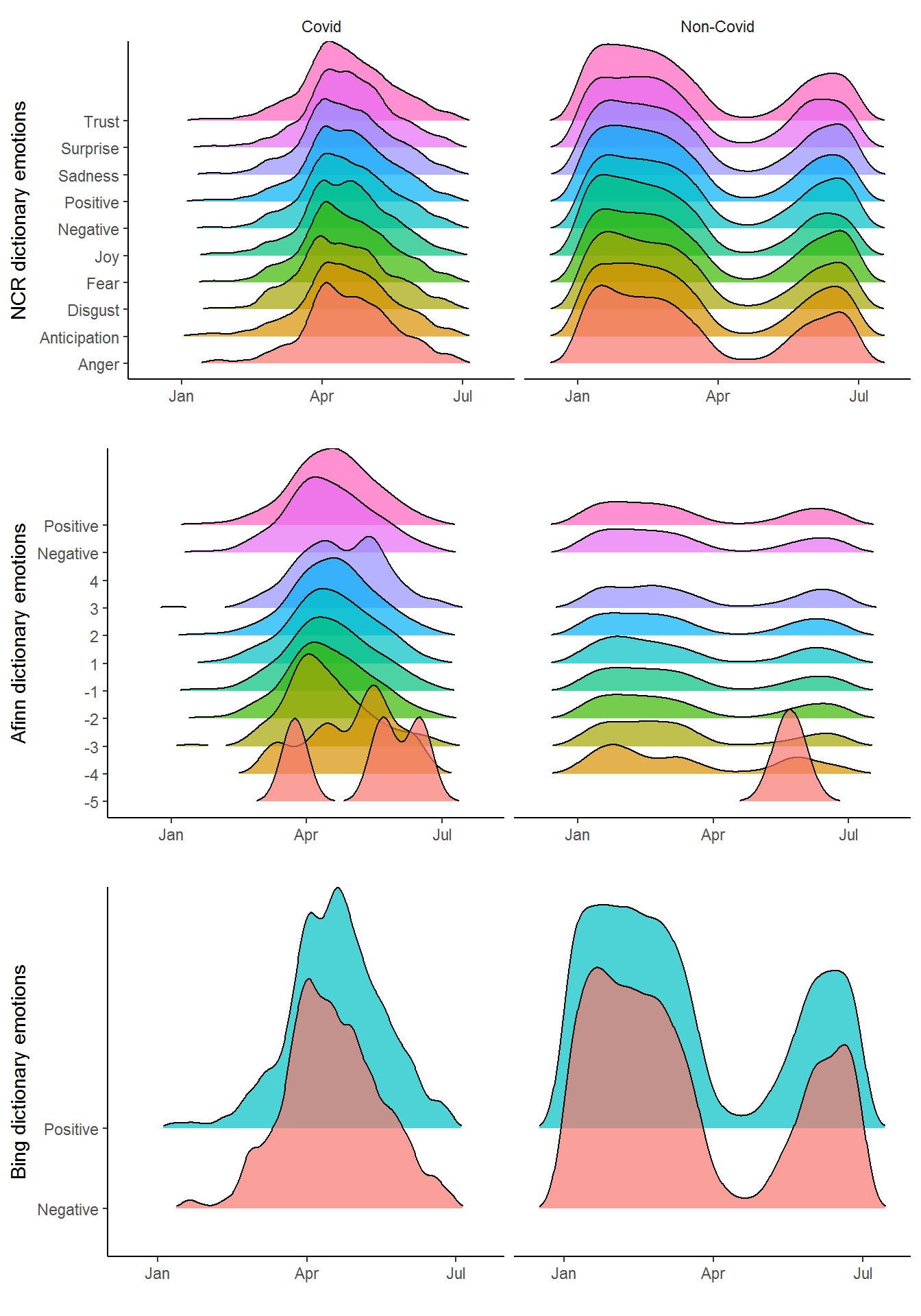

Supplement: Multimedia Appendix 4 [file medinform_v10i5e38308_app4.docx]

Multimedia Appendix 5. Distances between the time series of ENCN of Covid and non-Covid patients.


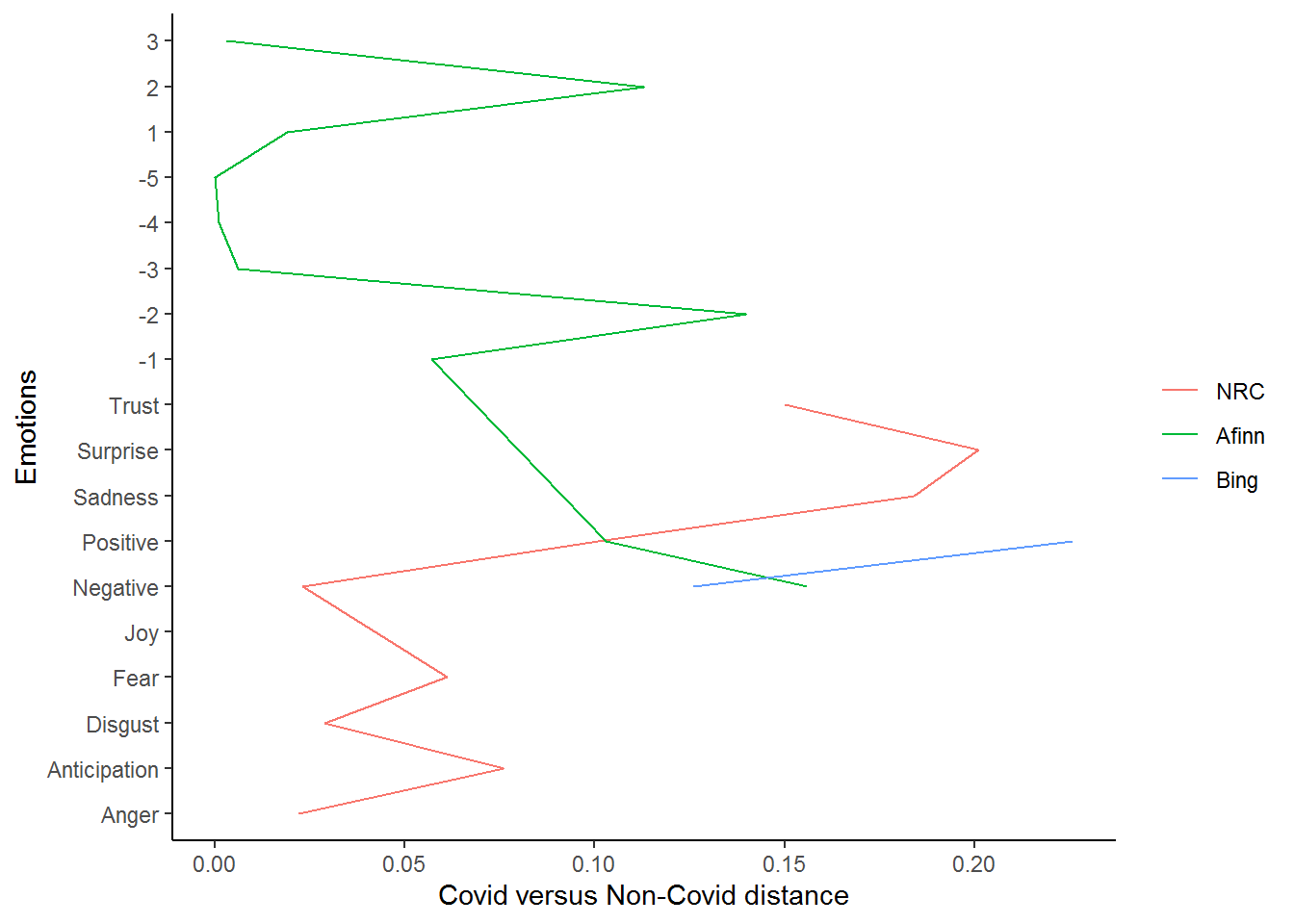

Supplement: Multimedia Appendix 5 [file medinform_v10i5e38308_app5.docx]
